# Heavy meson couplings in Hard-Wall AdS/QCD correspondence with *N*_*f*_ = 5

**DOI:** 10.1016/j.heliyon.2024.e25165

**Published:** 2024-01-26

**Authors:** S. Momeni, M. Saghebfar

**Affiliations:** a Department of Physics, Isfahan University, Iran; b Optics-Laser Science and Technology Research Center, Malek Ashtar University of Technology, Isfahan, Iran

## Abstract

In this article, Hard-Wall AdS/QCD with 5 flavors is used to study pseudoscalar, vector and axial-vector *B* meson. The mass and decay constants of $B^{{}_{0}}$, $B_{s}^{{}_{0}}$, $B_{c}^{{}_{-}}$, $\eta_{b}$, $B_{s}^{⁎0}$, $B_{c}^{{}_{⁎-}}$, $B_{1}^{{}_{-}}$, $B_{1s}^{{}_{0}}$, $B_{1c}^{{}_{-}}$, $\eta_{b}$ and $\chi_{{}_{b1}}$ as well as strong couplings of $\left({\overset{¯}{B}}^{{}_{⁎0}},B^{{}_{⁎+}},\pi^{-}\right)$$\left(B^{{}_{⁎+}},B_{s}^{{}_{⁎0}},K^{+}\right)$, $\left({\overset{¯}{B}}_{1}^{{}_{0}},B^{{}_{⁎+}},\pi^{-}\right)$, $\left(B_{1}^{{}_{⁎+}},B_{s}^{{}_{⁎0}},K^{+}\right)$, $\left({\overset{¯}{B}}_{1}^{{}_{0}},B_{1}^{{}_{+}},\rho^{-}\right)$, $\left({\overset{¯}{B}}_{1}^{{}_{+}},B_{s1}^{{}_{0}},K^{⁎+}\right)$, $\left({\overset{¯}{B}}_{1}^{{}_{0}},B^{{}_{⁎+}},a_{0}^{-}\right)$, $\left({\overset{¯}{B}}_{1}^{{}_{+}},B_{s}^{{}_{⁎0}},K_{0}^{+}\right)$, $\left({\overset{¯}{D}}^{{}_{⁎0}},D^{{}_{⁎-}},\pi^{-}\right)$, $\left({\overset{¯}{D}}^{{}_{⁎0}},D_{s}^{{}_{⁎-}},K^{+}\right)$$\left({\overset{¯}{D}}^{{}_{⁎0}},D_{1}^{{}_{-}},\pi^{-}\right)$, $\left({\overset{¯}{D}}^{{}_{⁎0}},D_{s1}^{{}_{-}},K^{+}\right)$, $\left({\overset{¯}{D}}_{1}^{{}_{0}},D_{1}^{{}_{-}},\rho^{-}\right)$, $\left({\overset{¯}{D}}_{1}^{{}_{0}},D_{s1}^{{}_{-}},K^{⁎+}\right)$, $\left({\overset{¯}{D}}^{{}_{⁎0}},D_{1}^{{}_{-}},a_{0}^{-}\right)$ and $\left({\overset{¯}{D}}^{{}_{⁎0}},D_{s1}^{{}_{-}},K_{0}^{+}\right)$ vertices are estimated in this study. A comparison of the results of the masses, decay constants, and strong couplings with existing predictions is also made.

## 1 Introduction

*B* and *D* mesons contain one bottom (*b*) and charm (*c*) quark (or anti-quark) and are classified in the heavy mesons category. Studying the heavy mesons is a valuable tool in phenomenological QCD to describe hadron interactions. We can obtain helpful information for considering the nature of these states by determining distribution amplitudes (DAs), wavefunction, decay constant, form factors of semileptonic decays and their couplings to the light mesons. Moreover, by obtaining the hadronic properties of the heavy mesons, we can investigate the final-state interactions, understand the production and absorption cross-sections in heavy-ion collisions, determine the Cabibbo-Kobayashi-Maskawa (CKM) matrix elements and explore new physics (NP) beyond the standard model (SM).

Strong coupling constants between mesons can help us to investigate the behavior of QCD at low energy and their evaluation includes non-perturbative QCD properties. So, some non-perturbative QCD approaches, such as QCD sum rules (QCDSR) and lattice QCD (LQCD) simulations are utilized to estimate *B* and *D* meson couplings to the light ones. The three-point QCD sum rules (3PSR) and the light-cone QCD sum rules (LCSR) approaches are according to the QCDSR method. The 3PSR model has been used to estimate $D^{⁎}D^{⁎}\rho$ [1], $D^{⁎}D\pi$, $B^{⁎}B\pi$ [2], [3], *DDρ* [4], $D^{⁎}D\rho$ [5], DDJ/ψ [6], $D^{⁎}DJ/\psi$ [7], $D^{⁎}D^{⁎}\pi$ [8], $D_{s}D^{⁎}K$, $D_{s}^{⁎}DK$ [9], *DDω* [10], $D_{s1}D^{⁎}K$, $D_{s1}D^{⁎}K_{0}^{⁎}$ [11], [12], $D_{s}D_{s}V$, $D_{s}^{⁎}D_{s}^{⁎}V$, $D_{s0}^{⁎}D_{s1}V$, $D_{s}D_{s}^{⁎}V$ [13], [14], $D_{1}D^{⁎}\pi$, $D_{1}D_{0}\pi$, $D_{1}D_{1}\pi$, $B_{1}B^{⁎}\pi$, $B_{1}B_{0}\pi$, $B_{1}B_{1}\pi$ [15], $D^{⁎}D^{⁎}J/\psi$ [16], $B_{s0}BK$ [17], $B_{s}^{⁎}BK$ [18], $D_{s}^{⁎}D_{s}\phi$ [19], $D_{s}DK_{0}^{⁎}$, $B_{s}BK_{0}^{⁎}$, $D_{s}^{⁎}DK$, $B_{s}^{⁎}BK$, $D_{s}^{⁎}DK_{1}$, $B_{s}^{⁎}BK_{1}$ [20], $D_{s}^{⁎}D^{⁎}K$, $D_{s1}D_{1}K^{⁎}$ [21], $D_{s}DK^{⁎}$, $D_{s}D^{⁎}K^{⁎}$ [22], $D^{⁎}D_{s}^{⁎}K$, $D_{1}D_{s1}K$, $D^{⁎}D_{s}K$, and $D_{1}D_{s0}^{⁎}K$ [23] vertices and the couplings of $D^{⁎}D_{s}K$, $D_{s}^{⁎}DK$, $D_{s0}DK$, $D_{0}D_{s}K$ [24], $D^{⁎}D^{⁎}P$, $D^{⁎}DV$, *DDV* [25], $DDA,D^{⁎}DA$ [26], $D_{\left(s\right)}^{⁎}D_{\left(s\right)}^{⁎}V$, $D_{s(1)}D_{s(1)}V$, $B_{\left(s\right)}^{⁎}B_{\left(s\right)}^{⁎}V$ and $B_{s(1)}B_{s(1)}V$ [27] are evaluated with the LCSR approach. Moreover, the coupling constants of $D^{⁎}\;D\;\pi$, $D^{⁎}\;D\;\gamma$, DDρ, $D^{⁎}\;D^{⁎}\;\rho$, $B^{⁎}\;B\;\pi$, vertices are studied via lattice QCD approach in [28], [29], [30], [31], [32], [33], [34].

In recent years, a relatively new a tool named the anti-de Sitter/quantum chromodynamics (AdS/QCD) correspondence has been developed by relating the non-perturbative quantities in 4-dimensional QCD to weakly coupled 5-dimensional gravitational theories on an anti-de Sitter AdS space [35], [36]. There are two models for describing the propagation of particles in the (AdS/QCD) theory: the hard-wall and soft-wall model. In the hard-wall model, the AdS space is compactified by two different boundary conditions on radial coordinate *z* as $\varepsilon (\to 0)\leq z\leq z_{0}$. The lower boundary on the *z* coordinate in the hard-wall model corresponds to the UV limit of QCD while $z_{0}$ simulates the confinement feature of QCD. On the other hand, in the soft-wall model, *z* coordinate varies from ≃0 up to infinity. However, the metric of AdS space is modified by a dilation factor, so the wavefunction becomes zero at infinity (more information is given in; [37], [38], [39], [40], [41], [42]). Using these two models with $N_{f}=3$, a wide range of phenomenological parameters such as masses, decay constants, electromagnetic and gravitational form factors, distribution amplitudes (DAs) for the light vector, axial vector, pseudoscalar mesons, and parton distribution functions (TDMs) are estimated in [37], [38], [39], [40], [41], [42], [43], [44], [45], [46], [47], [48], [49], [50], [51], [52], [53], [54], [55], [56], [57], [58], [59], [60], [61], [62], [63], [64], [65], [66], [67]. There are also studies of extending hard-wall holographic QCD to $N_{f}=4$ for the strong couplings of vertices $\left(\rho^{n},\rho ,\rho \right)$, $\left(\rho^{n},K,K\right)$, $\left(\rho^{n},K^{⁎},K^{⁎}\right)$, $\left(\rho^{n},D,D\right)$, $\left(\rho^{n},D^{⁎},D^{⁎}\right)$, $\left(D^{\left(⁎\right)},D,A\right)$, $\left(D^{\left(⁎\right)},D^{\left(⁎\right)},V\right)$, $\left(D_{1},D_{1},P\right)$, $\left(\psi ,D,D^{\left(⁎\right)},P\right)$, $\left(\psi ,D,D^{\left(⁎\right)},A\right)$ and the transition form factors of the semileptonic $D\to (V,A,S)\;\ell \;\nu_{\ell }$ decays in [68], [69], [70].

In the present paper, we will propose the framework of the Hard-Wall AdS/QCD with $N_{f}=5$ to study pseudoscalar, vector, and axial-vector *B* meson and estimate these states' masses, decay constants, and wavefunctions. Moreover, the strong couplings of $(B^{⁎},B^{⁎},P$), ($B_{1},B^{⁎},P$), ($B_{1},B_{1},V$), ($B_{1},B^{⁎},S$), ($D^{⁎},D^{⁎},P$), ($D_{1},D^{⁎},P$), ($D_{1},D_{1},V$) and ($D_{1},D^{⁎},S$) vertices will also be studied in our model. The paper is organized as follows: In Sec. 2, we have introduced our model, which contains scalar, pseudoscalar, vector, and axial vector mesons up to 5 quark flavors. This section extracts the wave functions and decay constants of considered mesons from our model. In Sec. 3, we derived details for the strong coupling constants of the said vertices. Sec. 4 describes numerical analysis for the mass, wavefunction, and decay constant of *B* mesons. This section also presents our estimation for the aforementioned couplings, and finally, the conclusions are stated in Sec. 5.

## 2 Mesons in Hard-Wall AdS/QCD model with $N_{f}=5$

To study the phenomenological quantities for the heavy or light mesons in the hard-wall AdS/QCD approach, we must obtain their equation of motion in 5 dimensional AdS space. Here the metric for $\mathrm{Ad}S_{5}$ is:

(1) $$ds^{2}=g_{MN}\;dx^{M}\;dx^{N}=\frac{R^{2}}{z^{2}}\;(\eta_{\mu \nu }dx^{\mu }\;dx^{\nu }-dz^{2}),\;\;\;\;(\varepsilon \leq z\leq z_{0}),$$

where $\eta_{\mu \nu }=\mathrm{diag}(1,-1,-1,-1)$ is the metric of the 4 dimensional flat space and *R* is the warp factor, which, in pure AdS, can be chosen as R=1 [35]. According to the general idea of the AdS/QCD, for every field in the $\mathrm{Ad}S_{5}$ space is included in our action, an operator is considered in 4-D QCD theory. The presented model in QCD side includes a scalar field *X* and $N_{f}$ gauge fields $L^{\mu ,a}$, $R^{\mu ,a}$ which are in correspondence with ${\overset{¯}{q}}_{L}\;q_{R}$, $J_{L}^{\mu ,a}={\overset{¯}{q}}_{L}\;\gamma^{\mu }\;t^{a}\;q_{L}$ and $J_{R}^{\mu ,a}={\overset{¯}{q}}_{R}\;\gamma^{\mu }\;t^{a}\;q_{R}$ in 5-D AdS space, respectively. Here, L(R) is used for the left-handed (right-handed) gauge, and *q* is the quark field. In the mentioned notations, $N_{f}$ is the number of flavors, and we take $N_{f}=5$ to describe all the light and the heavy (*D* and *B*) mesons using this model. Moreover, $q_{L/R}=(1\pm \gamma_{5})\;q$ and $t^{a}$ (with $a=1,\cdots N_{f}^{2}-1$) are the generators of $SU(N_{f})$. By following reference [35], the 5-D action with SU($N_{f}$)⊗L SU($N_{f}$)_*R*_ symmetry is as follows:

(2) $$S=\int d^{5}x\;\sqrt{g}\;\mathrm{Tr}\{(D_{M}X)\;{\left(D^{M}X\right)}^{†}+3{\left|X\right|}^{2}-\frac{1}{4g_{5}^{2}}\left(L^{MN}\;L_{MN}+R^{MN}\;R_{MN}\right)\},$$

where,

(3) $$D_{M}X=\partial_{M}X-iL_{M}X+iXR_{M},L_{MN}=\partial_{M}L_{N}-\partial_{N}L_{M}-i\left[L_{M},L_{N}\right],R_{MN}=\partial_{M}R_{N}-\partial_{N}R_{M}-i\left[R_{M},R_{N}\right].$$

Here, $L_{M}=L_{M}^{a}\;t^{a}$ and $R_{M}=R_{M}^{a}\;t^{a}$. Using the right-hand gauge fields, vector (V) and axial vector (A) can be written as V=(L+R)/2 and A=(L−R)/2. The *X* field can also be written in terms of a classical part $X_{0}$ and two exponential factors as:

(4) $$X(x,z)=e^{i\pi^{a}(x,z)t^{a}}\;X_{0}(z)\;e^{i\pi^{a}(x,z)t^{a}}.$$

In this equation *π* contains the fluctuations. By solving the equation of motion of $X_{0}(z)$, one obtains:

(5) $$2X_{0ij}(z)=\zeta M_{ij}\;z+\frac{\Sigma_{ij}}{\zeta }\;z^{3},$$

where *M* is the quark-mass matrix, and the quark condensates matrix is denoted with Σ. According to [71], [72] $\zeta =\sqrt{N_{c}}/2\pi$ is a rescaling parameter. With $N_{f}=5$, we consider $M=\mathrm{diag}(m_{u},m_{d},m_{s},m_{c},m_{b})$ and $\Sigma =\mathrm{diag}(\sigma_{u},\sigma_{d},\sigma_{s},\sigma_{c},\sigma_{b})$, and now it's time to extract wavefunctions from this model. First, the action is given in Eq. (2) must be expanded up to the second order in terms of vector (V), axial vector (A), and pseudoscalar field (*π*). With considering $g^{MN}=z^{2}\;\eta^{MN}$, we have [51]:

(6) $$S=\int \;d^{5}x\{\sum_{a=1}^{15}\frac{-1}{4g_{5}^{2}z}\eta^{MM^{\prime }}\;\eta^{NN^{\prime }}\;(\partial_{M}V_{N}^{a}-\partial_{N}V_{M}^{a})\;(\partial_{M^{\prime }}V_{N^{\prime }a}-\partial_{N^{\prime }}V_{M^{\prime }a})+\frac{{M_{V}^{a}}^{2}}{2z^{3}}\eta^{MM^{\prime }}V_{Ma}V_{M^{\prime }}^{a}\;\frac{-1}{4g_{5}^{2}z}\eta^{MM^{\prime }}\;\eta^{NN^{\prime }}(\partial_{M}A_{N}^{a}-\partial_{N}A_{M}^{a})\;(\partial_{M^{\prime }}A_{N^{\prime }a}-\partial_{N^{\prime }}A_{M^{\prime }a})+\frac{{M_{A}^{a}}^{2}}{2z^{3}}\eta^{MM^{\prime }}(\partial_{M}\pi^{a}-A_{M}^{a})\;(\partial_{M^{\prime }}\pi_{b}-A_{M^{\prime }b})\}.$$

The following two definitions are inspired by reference [51], to calculate the mass combinations:

(7) $${M_{V}^{a}}^{2}\delta^{ab}=-2\;\mathrm{Tr}\left([t^{a},X_{0}][t^{b},X_{0}\right]),{M_{A}^{a}}^{2}\delta^{ab}=2\;\mathrm{Tr}\left(\{t^{a},X_{0}\}\{t^{b},X_{0}\right\}).$$

After performing the relevant calculations, considering (a=1,⋯,24) and $v_{q}(z)=\zeta m_{q}z+\frac{1}{\zeta }\sigma_{q}z^{3}$ with q=(u,d,s,c,b), the following values for ${M_{V}^{a}}^{2}$ and ${M_{A}^{a}}^{2}$ are obtained, which are written in Table 1.

**Table 1:** Calculated values of ${M_{V}^{a}}^{2}$ and ${M_{A}^{a}}^{2}$ for (*a* = 1,⋯,24) and $v_{q}(z)=\zeta m_{q}z+\frac{1}{\zeta }\sigma_{q}z^{3}$ with *q* = (*u*,*d*,*s*,*c*,*b*).

*a*	${M_{V}^{a}}^{2}$	${M_{A}^{a}}^{2}$	*a*	${M_{V}^{a}}^{2}$	${M_{A}^{a}}^{2}$
(1,2)	$\frac{1}{4}{\left(v_{u}-v_{d}\right)}^{2}$	$\frac{1}{4}{\left(v_{u}+v_{d}\right)}^{2}$	(13,14)	$\frac{1}{4}{\left(v_{d}-v_{c}\right)}^{2}$	$\frac{1}{4}{\left(v_{d}+v_{c}\right)}^{2}$
3	0	$\frac{1}{2}(v_{u}^{2}+v_{d}^{2})$	(15,16)	$\frac{1}{4}{\left(v_{d}-v_{b}\right)}^{2}$	$\frac{1}{4}{\left(v_{d}+v_{b}\right)}^{2}$
(4,5)	$\frac{1}{4}{\left(v_{u}-v_{s}\right)}^{2}$	$\frac{1}{4}{\left(v_{u}+v_{s}\right)}^{2}$	(17,18)	$\frac{1}{4}{\left(v_{s}-v_{c}\right)}^{2}$	$\frac{1}{4}{\left(v_{s}+v_{c}\right)}^{2}$
(6,7)	$\frac{1}{4}{\left(v_{d}-v_{s}\right)}^{2}$	$\frac{1}{4}{\left(v_{d}+v_{s}\right)}^{2}$	(19,20)	$\frac{1}{4}{\left(v_{s}-v_{b}\right)}^{2}$	$\frac{1}{4}{\left(v_{s}+v_{b}\right)}^{2}$
8	0	$\frac{1}{6}(v_{u}^{2}+v_{d}^{2}+4v_{s}^{2})$	(21,22)	$\frac{1}{4}{\left(v_{c}-v_{b}\right)}^{2}$	$\frac{1}{4}{\left(v_{c}+v_{b}\right)}^{2}$
(9,10)	$\frac{1}{4}{\left(v_{u}-v_{c}\right)}^{2}$	$\frac{1}{4}{\left(v_{u}+v_{c}\right)}^{2}$	23	0	$\frac{1}{2}(v_{c}^{2}+v_{b}^{2})$
(11,12)	$\frac{1}{4}{\left(v_{u}-v_{b}\right)}^{2}$	$\frac{1}{4}{\left(v_{u}+v_{b}\right)}^{2}$	24	0	$\frac{2}{15}(v_{u}^{2}+v_{d}^{2}+v_{s}^{2}+\frac{9}{4}v_{c}^{2}+\frac{9}{4}v_{b}^{2})$

The formalism for extracting wavefunctions and decay constants of vector, axial vector, pseudoscalar, and scalar mesons from Eq. (6) can be found in [51], [52], [55], [69], [70]. Starting from the equations of motion for the vector field $\left(V_{M}^{a}\right)$, and the axial vector field $\left(A_{M}^{a}\right)$, we can write [51]:

(8) $$\eta^{ML}\partial_{M}\left(\frac{1}{z}\left(\partial_{L}V_{N}^{a}-\partial_{N}V_{L}^{a}\right)\right)+\frac{\alpha^{a}(z)}{z}V_{N}^{a}=0,$$

(9) $$\eta^{ML}\partial_{M}\left(\frac{1}{z}\left(\partial_{L}A_{N}^{a}-\partial_{N}A_{L}^{a}\right)\right)+\frac{\beta^{a}(z)}{z}A_{N}^{a}=0.$$

Here, we have defined: $z^{2}\alpha^{a}(z)=g_{5}^{2}\;{M_{V}^{a}}^{2}$ and $z^{2}\beta^{a}(z)=g_{5}^{2}\;{M_{A}^{a}}^{2}$. If, $V_{\mu ⊥}^{a}$ and $A_{\mu ⊥}^{a}$ describe vector and axial vector states, then considering $V_{N}^{a}=(V_{\mu }^{a},V_{z}^{a})$ and $A_{N}^{a}=(A_{\mu }^{a},A_{z}^{a})$ and choosing the gauge $A_{z}^{a}=0$ where $V_{\mu }^{a}=V_{\mu ⊥}^{a}+V_{\mu ∥}^{a}$ and $A_{\mu }^{a}=A_{\mu ⊥}^{a}+A_{\mu ∥}^{a}$, we will reach the following equations of motion [51]:

(10) $$\left(\partial_{z}\frac{1}{z}\partial_{z}+\frac{q^{2}-\alpha^{a}}{z}\right)V_{\mu ⊥}^{a}(q,z)=0,$$

(11) $$\left(\partial_{z}\frac{1}{z}\partial_{z}+\frac{q^{2}-\beta^{a}}{z}\right)A_{\mu ⊥}^{a}(q,z)=0,$$

where *q* is the Fourier variable conjugate to the 4-dimensional coordinates, *x*. It should be noted that to find Eqs. (10), (11), the choices $\partial^{\mu }V_{\mu ⊥}^{a}(x,z)=0$ and $\partial^{\mu }A_{\mu ⊥}^{a}(x,z)=0$ are applied in the equations of motion. The transverse part of the vector field and the axial vector can be expressed by an equation $F_{\mu ⊥}^{a}(q,z)=F_{\mu ⊥}^{0a}(q)F^{a}(q^{2},z)$ that if F=V, it represents transverse part of the vector field, and if F=A, it denotes axial vector. $F_{\mu ⊥}^{0a}$ and $F^{a}(q^{2},z)$ are boundary values at UV and bulk-to-boundary propagator for $F_{\mu ⊥}^{a}$, respectively. Bulk-to-boundary propagators $F^{a}(q^{2},z)$ (for F=(V,A)) satisfy the same equation as $F_{\mu ⊥}^{a}(q,z)$ with the boundary conditions $F^{a}(q^{2},\varepsilon )=1$ and $\partial_{z}F^{a}(q^{2},z_{0})=0$. The longitudinal parts of the vector field, which describe the scalar states, are defined as $V_{\mu ∥}^{a}=\partial_{\mu }\xi^{a}$ and $V_{z}^{a}=-\partial_{z}{\overset{˜}{\pi }}^{a}$, where $\xi^{a}={\overset{˜}{\phi }}^{a}-{\overset{˜}{\pi }}^{a}$, and these two parts are coupled in a system of differential equations as follows [51]:

(12) $$-q^{2}\partial_{z}{\overset{˜}{\phi }}^{a}(q^{2},z)+\alpha^{a}\partial_{z}{\overset{˜}{\pi }}^{a}(q^{2},z)=0\;,$$

(13) $$\partial_{z}(\frac{1}{z}\partial_{z}{\overset{˜}{\phi }}^{a}(q^{2},z))-\frac{\alpha^{a}}{z}({\overset{˜}{\phi }}^{a}(q^{2},z)-{\overset{˜}{\pi }}^{a}(q^{2},z))=0.$$

The boundary conditions for ${\overset{˜}{\phi }}^{a}$ and ${\overset{˜}{\pi }}^{a}$ are ${\overset{˜}{\phi }}^{a}(q,\varepsilon )=0$, ${\overset{˜}{\pi }}^{a}(q,\varepsilon )=-1$ and $\partial_{z}{\overset{˜}{\phi }}^{a}(q^{2},z_{0})=\partial_{z}{\overset{˜}{\pi }}^{a}(q^{2},z_{0})=0$. At this point, the only remaining states of mesons are the states related to pseudoscalar mesons, which $\pi^{a}$ and $\phi^{a}$ with $A_{\mu ∥}^{a}=\partial_{\mu }\phi^{a}$, describe these states as following coupled equations [51]:

(14) $$-q^{2}\partial_{z}\phi^{a}(q^{2},z)+\beta^{a}(z)\partial_{z}\pi^{a}(q^{2},z)=0\;,$$

(15) $$\partial_{z}\left(\frac{1}{z}\partial_{z}\phi^{a}(q^{2},z)\right)-\frac{\beta^{a}(z)}{z}\left(\phi^{a}(q^{2},z)-\pi^{a}(q^{2},z)\right)=0\;.$$

The boundary conditions for solving Eqs. (14), (15) are $\phi^{a}(q^{2},\varepsilon )=0$, $\pi^{a}(q^{2},\varepsilon )=-1$, and $\partial_{z}\phi^{a}(q^{2},z_{0})=\partial_{z}\pi^{a}(q^{2},z_{0})=0$.

In order to solve Eqs. (10)-(15), Green's function formalism is used, where $V^{a}$, $A^{a}$, $\pi^{a}(\phi^{a})$ and ${\overset{˜}{\pi }}^{a}({\overset{˜}{\phi }}^{a})$ can be written as a sum over the vector (*V*), axial vector (*A*), pseudoscalar (*P*), and scalar (*S*) mesons poles as follows [51]:

(16) $$V^{a}(q^{2},z)=\sum_{n}\frac{-g_{5}f_{{}_{V^{n}}}^{a}\psi_{{}_{V^{n}}}^{a}(z)}{q^{2}-m_{{}_{V^{n}}}^{2}}\;,\;\;\;A^{a}(q^{2},z)=\sum_{n}\frac{-g_{5}f_{{}_{A^{n}}}^{a}\psi_{{}_{A^{n}}}^{a}(z)}{q^{2}-m_{{}_{A^{n}}}^{a2}}\;,$$

(17) $$\phi^{a}(q^{2},z)=\sum_{n}\frac{-g_{5}m_{{}_{P^{n}}}^{a2}f_{{}_{P^{n}}}^{a}\phi_{n}^{a}(z)}{q^{2}-m_{{}_{P^{n}}}^{a2}}\;,\;\;\;\pi^{a}(q^{2},z)=\sum_{n}\frac{-g_{5}m_{{}_{P^{n}}}^{a2}f_{{}_{P^{n}}}^{a}\pi_{n}^{a}(z)}{q^{2}-m_{{}_{P^{n}}}^{a2}}\;,$$

(18) $${\overset{˜}{\phi }}^{a}(q^{2},z)=\sum_{n}\frac{-g_{5}m_{{}_{S^{n}}}^{a2}f_{{}_{S^{n}}}^{a}{\overset{˜}{\phi }}_{n}^{a}(z)}{q^{2}-m_{{}_{S^{n}}}^{a2}}\;,\;\;\;{\overset{˜}{\pi }}^{a}(q^{2},z)=\sum_{n}\frac{-g_{5}m_{{}_{S^{n}}}^{a2}f_{{}_{S^{n}}}^{a}{\overset{˜}{\pi }}_{n}^{a}(z)}{q^{2}-m_{{}_{S^{n}}}^{a2}}.$$

Where $f_{{}_{V{\left(A\right)}^{n}}}^{\frac{1}{2}}$ and $f_{{}_{P{\left(S\right)}^{n}}}$ are decay constants of the $n^{th}$ mode of vector (axial vector) and pseudoscalar (scalar) mesons and are obtained according to the following equations [35]:

(19) $$f_{{}_{V^{n}}}^{a}=\frac{\partial_{z}\psi_{{}_{V^{n}}}^{a}}{g_{5}\;z}{|}_{z=\epsilon },\;\;\;f_{{}_{A^{n}}}^{a}=\frac{\partial_{z}\psi_{{}_{A^{n}}}^{a}}{g_{5}\;z}{|}_{z=\epsilon },$$

(20) $$f_{{}_{P^{n}}}^{a}=-\frac{\partial_{z}\phi_{n}^{a}}{g_{5}\;z}{|}_{z=\epsilon },\;\;\;f_{{}_{S^{n}}}^{a}=-\frac{\partial_{z}{\overset{˜}{\phi }}_{n}^{a}}{g_{5}\;z}{|}_{z=\epsilon }.$$

Finally, after explaining the theoretical aspects, the dependence of SU(5) Vector (*V*), axial vector (*A*), pseudoscalar (*π*), and scalar (*S*) mesons matrices in terms of the charged states can be expressed as follows:

$$V=\frac{1}{\sqrt{2}}\left(\begin{array}{ccccc} \frac{\rho^{0}}{\sqrt{2}}+\frac{\omega^{\prime }}{\sqrt{6}}-\frac{2}{\sqrt{30}}ϒ & \rho^{{}_{+}} & K^{{}_{⁎+}} & {\overset{¯}{D}}^{{}_{⁎0}} & B^{{}_{⁎+}}\\ \rho^{{}_{-}} & -\frac{\rho^{0}}{\sqrt{2}}+\frac{\omega^{\prime }}{\sqrt{6}}-\frac{2}{\sqrt{30}}ϒ & K^{{}_{⁎0}} & D^{{}_{⁎-}} & B^{{}_{⁎0}}\\ K^{{}_{⁎-}} & {\overset{¯}{K}}^{{}_{⁎0}} & -\sqrt{\frac{2}{3}}\omega^{\prime }-\frac{2}{\sqrt{30}}ϒ & D_{s}^{{}_{⁎-}} & B_{s}^{{}_{⁎0}}\\ D^{{}_{⁎0}} & D^{{}_{⁎+}} & D_{s}^{{}_{⁎+}} & \frac{\psi }{\sqrt{2}}+\frac{3}{\sqrt{30}}ϒ & B_{c}^{{}_{⁎+}}\\ B^{{}_{⁎+}} & {\overset{¯}{B}}^{{}_{⁎0}} & {\overset{¯}{B}}_{s}^{{}_{⁎0}} & B_{c}^{{}_{⁎-}} & -\frac{\psi }{\sqrt{2}}+\frac{3}{\sqrt{30}}ϒ \end{array}\right),$$

$$A=\frac{1}{\sqrt{2}}\left(\begin{array}{ccccc} \frac{a_{1}^{0}+b_{1}^{0}}{\sqrt{2}}+\frac{f_{1}+f_{1}^{\prime }}{\sqrt{6}}-\frac{2\;\chi_{{}_{b1}}}{\sqrt{30}}\; & a_{1}^{{}_{+}}+b_{1}^{{}_{+}}\; & K_{1A}^{{}_{+}}+K_{1B}^{{}_{+}}\; & {\overset{¯}{D}}_{1}^{{}_{0}}\; & B_{1}^{{}_{+}}\\ a_{1}^{{}_{-}}+b_{1}^{{}_{-}}\; & -\frac{a_{1}^{{}_{0}}+b_{1}^{{}_{0}}}{\sqrt{2}}+\frac{f_{1}+f_{1}^{\prime }}{\sqrt{6}}-\frac{2\;\chi_{{}_{b1}}}{\sqrt{30}}\; & K_{1A}^{{}_{0}}+K_{1B}^{{}_{0}}\; & D_{1}^{{}_{-}}\; & B_{1}^{{}_{0}}\\ K_{1A}^{{}_{-}}+K_{1B}^{{}_{-}}\; & {\overset{¯}{K}}_{1A}^{{}_{0}}+{\overset{¯}{K}}_{1B}^{{}_{0}}\; & -\sqrt{\frac{2}{3}}(f_{1}+f_{1}^{\prime })-\frac{2\;\chi_{{}_{b1}}}{\sqrt{30}}\; & D_{s1}^{{}_{-}}\; & B_{1s}^{{}_{0}}\\ D_{1}^{{}_{0}}\; & D_{1}^{{}_{+}}\; & D_{s1}^{{}_{+}}\; & \frac{\chi_{{}_{c1}}}{\sqrt{2}}+\frac{3\;\chi_{{}_{b1}}}{\sqrt{30}}\; & B_{1c}^{{}_{+}}\\ B_{1}^{{}_{-}}\; & {\overset{¯}{B}}_{1}^{{}_{0}}\; & {\overset{¯}{B}}_{1s}^{{}_{0}}\; & B_{1c}^{{}_{-}}\; & -\frac{\chi_{{}_{c1}}}{\sqrt{2}}+\frac{3\;\chi_{{}_{b1}}}{\sqrt{30}} \end{array}\right),$$

$$\pi =\frac{1}{\sqrt{2}}\left(\begin{array}{ccccc} \frac{\pi^{{}_{0}}}{\sqrt{2}}+\frac{\eta }{\sqrt{6}}-\frac{2}{\sqrt{30}}\eta_{b} & \pi^{{}_{+}} & K^{{}_{+}} & {\overset{¯}{D}}^{{}_{0}} & B^{{}_{+}}\\ \pi^{{}_{-}} & -\frac{\pi^{0}}{\sqrt{2}}+\frac{\eta }{\sqrt{6}}-\frac{2}{\sqrt{30}}\eta_{b} & K^{{}_{0}} & D^{{}_{-}} & B^{{}_{0}}\\ K^{{}_{-}} & {\overset{¯}{K}}^{{}_{0}} & -\sqrt{\frac{2}{3}}\eta -\frac{2}{\sqrt{30}}\eta_{b} & D_{s}^{{}_{-}} & B_{s}^{{}_{0}}\\ D^{{}_{0}} & D^{{}_{+}} & D_{s}^{{}_{+}} & \frac{\eta_{c}}{\sqrt{2}}+\frac{3}{\sqrt{30}}\eta_{b} & B_{c}^{{}_{+}}\\ B^{{}_{-}} & {\overset{¯}{B}}^{{}_{0}} & {\overset{¯}{B}}^{{}_{0}} & B_{c}^{{}_{-}} & -\frac{\eta_{c}}{\sqrt{2}}+\frac{3}{\sqrt{30}}\eta_{b} \end{array}\right),$$

$$S=\frac{1}{\sqrt{2}}\left(\begin{array}{ccccc} \frac{a_{0}^{{}_{0}}}{\sqrt{2}}+\frac{\sigma }{\sqrt{6}}-\frac{2}{\sqrt{30}}\chi_{{}_{b0}} & a_{0}^{{}_{+}} & K_{0}^{{}_{+}} & {\overset{¯}{D}}_{{}_{0}} & B_{0}^{{}_{+}}\\ a_{0}^{{}_{-}} & -\frac{a_{0}^{{}_{0}}}{\sqrt{2}}+\frac{\sigma }{\sqrt{6}}-\frac{2}{\sqrt{30}}\chi_{{}_{b0}} & K_{0} & D_{0}^{{}_{-}} & B_{0}\\ K_{0}^{{}_{-}} & {\overset{¯}{K}}_{{}_{0}} & -\sqrt{\frac{2}{3}}\sigma -\frac{2}{\sqrt{30}}\chi_{{}_{b0}} & D_{0s}^{{}_{-}} & B_{{}_{0s}}\\ D_{{}_{0}} & D_{0}^{{}_{+}} & D_{0s}^{{}_{+}} & \frac{\chi_{{}_{c0}}}{\sqrt{2}}+\frac{3}{\sqrt{30}}\chi_{{}_{b0}} & B_{{}_{0c}}^{{}_{+}}\\ B_{0}^{{}_{-}} & {\overset{¯}{B}}_{0} & {\overset{¯}{B}}_{{}_{0s}} & B_{{}_{0c}}^{{}_{-}} & -\frac{\chi_{{}_{c0}}}{\sqrt{2}}+\frac{3}{\sqrt{30}}\chi_{{}_{b0}} \end{array}\right).$$

## 3 Strong coupling constants

In this section, the couplings of $(B^{⁎},B^{⁎},P$), ($B_{1},B^{⁎},P$), ($B_{1},B_{1},V$), and ($B_{1},B^{⁎},S$) vertices are derived in the Hard-Wall AdS/QCD model according to the following definitions [73], [74], [75]:

(21) $$\langle B^{⁎}(p_{1},\varepsilon_{{}_{1}})|B^{⁎}(p_{2},\varepsilon_{{}_{2}})\;P(p_{3})\rangle =[(\varepsilon_{{}_{1}}^{⁎}.\varepsilon_{{}_{2}})\;(p_{3}.p_{1})-(\varepsilon_{{}_{1}}^{⁎}.p_{3})\;(\varepsilon_{{}_{2}}.p_{1})]\;g_{{}_{B^{⁎}\;B^{⁎}\;P}},\langle B_{1}(p_{1},\varepsilon^{\prime })|B^{⁎}(p_{2},\varepsilon )\;P(p_{3})\rangle =[(\varepsilon^{\prime \;⁎}.\varepsilon )\;(p_{3}.p_{1})-(\varepsilon^{\prime \;⁎}.p_{3})\;(\varepsilon .p_{1})]\;g_{{}_{B_{1}\;B^{⁎}\;P}},\langle B_{1}(p_{1},\varepsilon_{1}^{\prime })|B_{1}(p_{2},\varepsilon_{2}^{\prime })\;V(p_{3},\varepsilon )\rangle =[(p_{1}.\varepsilon_{2}^{\prime })\;(\varepsilon_{{}_{1}}^{\prime \;⁎}.\varepsilon )-(p_{2}.\varepsilon_{{}_{1}}^{\prime \;⁎})\;(\varepsilon_{2}^{\prime }.\varepsilon )]\;g_{{}_{B_{1}\;B_{1}\;V}},\langle B_{1}(p_{1},\varepsilon^{\prime })|B^{⁎}(p_{2},\varepsilon )\;S(p_{3})=[(\varepsilon^{\prime \;⁎}.\varepsilon )\;(p_{3}.p_{1})-(\varepsilon^{\prime \;⁎}.p_{3})\;(\varepsilon .p_{1})]\;g_{{}_{B_{1}\;B^{⁎}\;S}}.$$

**Figure 1:**
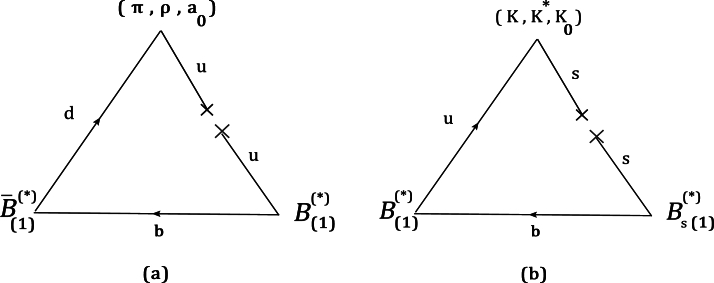
3-particle diagrams for (*B*^⁎^,*B*^⁎^,*P*), (*B*_1_,*B*^⁎^,*P*), (*B*_1_,*B*_1_,*V*), and (*B*_1_,*B*^⁎^,*S*) vertices. Diagram (a): vertices $\left({\overset{¯}{B}}^{{}_{⁎0}},B^{{}_{⁎+}},\pi^{-}\right)$, $\left({\overset{¯}{B}}_{1}^{{}_{0}},B^{{}_{⁎+}},\pi^{-}\right)$, $\left({\overset{¯}{B}}_{1}^{{}_{0}},B_{1}^{{}_{+}},\rho^{-}\right)$, and $\left({\overset{¯}{B}}_{1}^{{}_{0}},B^{{}_{⁎+}},a_{0}^{-}\right)$. Diagram (b): vertices $\left(B^{{}_{⁎+}},B_{s}^{{}_{⁎0}},K^{+}\right)$, $\left(B_{1}^{{}_{⁎+}},B_{s}^{{}_{⁎0}},K^{+}\right)$, $\left({\overset{¯}{B}}_{1}^{{}_{+}},B_{s1}^{{}_{0}},K^{⁎+}\right)$, and $\left({\overset{¯}{B}}_{1}^{{}_{+}},B_{s}^{{}_{⁎0}},K_{0}^{+}\right)$.

In the mentioned equations, $p_{1}=p_{2}+p_{3}$ and the corresponding diagrams for these vertices are given in Fig. 1 (a,b). The vertices $\left({\overset{¯}{B}}^{{}_{⁎0}},B^{{}_{⁎+}},\pi^{-}\right)$, $\left({\overset{¯}{B}}_{1}^{{}_{0}},B^{{}_{⁎+}},\pi^{-}\right)$, $\left({\overset{¯}{B}}_{1}^{{}_{0}},B_{1}^{{}_{+}},\rho^{-}\right)$, and $\left({\overset{¯}{B}}_{1}^{{}_{0}},B^{{}_{⁎+}},a_{0}^{-}\right)$ can be described with diagram (a), while diagram (b) is used to explain $\left(B^{{}_{⁎+}},B_{s}^{{}_{⁎0}},K^{+}\right)$, $\left(B_{1}^{{}_{⁎+}},B_{s}^{{}_{⁎0}},K^{+}\right)$, $\left({\overset{¯}{B}}_{1}^{{}_{+}},B_{s1}^{{}_{0}},K^{⁎+}\right)$, and $\left({\overset{¯}{B}}_{1}^{{}_{+}},B_{s}^{{}_{⁎0}},K_{0}^{+}\right)$ vertices.

To obtain couplings of these vertices in the Hard-Wall AdS/QCD, the 3-point correlation functions, including the currents of 3 involving particles, are needed. Utilizing AdS/QCD correspondence, these correlation functions can be obtained by functionally differentiating the 5-D action with respect to their sources [45], [51], [76], [77]. For considered vertices, the 3-point correlation functions can be written as the following relations:

(22) $$\langle 0|T\left\{J_{V⊥}^{\mu a}(x)J_{V⊥}^{\nu b}(y)J_{A∥}^{\alpha c}(w\right)\}|0\rangle =-\frac{\;\delta^{3}S(B^{⁎}\;B^{⁎}\;P)\;}{\delta V_{⊥\mu }^{0a}(x)\;\delta V_{⊥\nu }^{0b}(y)\;\delta A_{∥\alpha }^{0c}(w)}\;\;\;\;\;\;(\text{for}\;\;B^{⁎}\;B^{⁎}\;P\;\;\text{vertex}),$$

(23) $$\langle 0|T\left\{J_{A⊥}^{\mu a}(x)J_{V⊥}^{\nu b}(y)J_{A∥}^{\alpha c}(w\right)\}|0\rangle =-\frac{\;\delta^{3}S(B_{1}\;B^{⁎}\;P)\;}{\delta A_{⊥\mu }^{0a}(x)\;\delta V_{⊥\nu }^{0b}(y)\;\delta A_{∥\alpha }^{0c}(w)}\;\;\;\;\;\;(\text{for}\;\;B_{1}\;B^{⁎}\;P\;\;\text{vertex}),$$

(24) $$\langle 0|T\left\{J_{A⊥}^{\mu a}(x)J_{A⊥}^{\nu b}(y)J_{V⊥}^{\sigma c}(w\right)\}|0\rangle =-\frac{\;\delta^{3}S(B_{1}\;B_{1}\;V)\;}{\delta A_{⊥\mu }^{0a}(x)\;\delta A_{⊥\nu }^{0b}(y)\;\delta V_{⊥\sigma }^{0c}(w)}\;\;\;\;\;\;(\text{for}\;\;B_{1}\;B_{1}\;V\;\;\text{vertex}),$$

(25) $$\langle 0|T\left\{J_{A⊥}^{\mu a}(x)J_{V⊥}^{\nu b}(y)J_{V∥}^{\alpha c}(w\right)\}|0\rangle =-\frac{\;\delta^{3}S(B_{1}\;B^{⁎}\;S)\;}{\delta A_{⊥\mu }^{0a}(x)\;\delta V_{⊥\nu }^{0b}(y)\;\delta V_{∥\alpha }^{0c}(w)}\;\;\;\;\;\;(\text{for}\;\;B_{1}\;B^{⁎}\;S\;\;\text{vertex}).$$

Here, for (1,2,3) vertex, the relevant action S(123) is separated from the total 5-D action. For example, for $\left(B_{1}\;B^{⁎}\;P\right)$, we must consider the parts, which include one axial vector and one vector pseudoscalar state. In this step, we obtain:

(26) $$S(B^{⁎}\;B^{⁎}\;P)=\int d^{5}x(\frac{k^{abc}}{z^{3}}[V_{\mu }^{a}\;V^{\mu b}\;\pi^{c}+V_{z}^{a}\;V^{zb}\;\pi^{c}]),$$

(27) $$S(B_{1}\;B^{⁎}\;P)=\int d^{5}x(\frac{f^{abc}}{2\;g_{5}^{2}\;z}[A^{\mu a}\;V_{\mu \nu }^{b}\;\partial^{\nu }\phi^{c}]+\frac{h^{abc}}{z^{3}}[A_{\mu }^{a}\;V^{b\mu }\;\pi^{c}]+\frac{g^{abc}}{z^{3}}[A_{\mu }^{a}\;V^{b\mu }\;\pi^{c}])$$

(28) $$S(B_{1}\;B_{1}\;V)=\int d^{5}x(\frac{f^{abc}}{2g_{5}^{2}z}[V_{\mu \nu }^{a}\;A^{\mu b}\;V^{\nu c}+A_{\mu \nu }^{a}\;V^{\mu b}\;A^{\nu c}]),$$

(29) $$S(B_{1}\;B^{⁎}\;S)=\int d^{5}x(-\frac{f^{abc}}{g_{5}^{2}\;z}[A_{\mu \nu }^{a}\;V^{\mu b}\;\partial^{\nu }\xi^{c}-\partial_{z}A_{\mu }^{a}\;V^{\mu b}\;\partial_{z}{\overset{˜}{\pi }}^{c}])+\int d^{5}x(\frac{f^{abc}}{g_{5}^{2}\;z}[V_{\mu \nu }^{a}\;A^{\mu b}\;\partial^{\nu }\xi^{c}+\partial_{z}V_{\mu }^{a}\;A^{\mu b}\;\partial_{z}{\overset{˜}{\pi }}^{c}]),$$

where $f^{abc}$ is the structure constant of $S(U_{5})$ and $k^{abc}$, $g^{abc}$, and $h^{abc}$ are obtained from the following equations:

(30) $$\;\;\;k^{abc}=-2i\;\mathrm{Tr}\;(\left[t^{a},X_{0}\right]\left[t^{b},\left\{t^{c},X_{0}\right\}\right]),$$

(31) $$\;\;\;\;g^{abc}=-2i\;\mathrm{Tr}\;(\left\{t^{a},X_{0}\right\}\left[t^{b},\left\{t^{c},X_{0}\right\}\right]),$$

(32) $$h^{abc}=-2i\;\mathrm{Tr}\;(\left[t^{a},X_{0}\right]\left\{t^{b},\left\{t^{c},X_{0}\right\}\right\}).$$

To calculate the couplings of considered vertices, we insert three complete sets of intermediate states with the same quantum numbers as the meson currents into the correlation function and use the definitions of the decay constant of vector, axial-vector, pseudoscalar, and the scalar mesons as follows [51], [52]:

(33) $$\langle 0|J_{V⊥}^{\rho a}|V^{a^{\prime }}(p,\varepsilon_{{}_{V}})\rangle =f_{{}_{V}}\;\varepsilon^{\rho }\;\delta^{aa^{\prime }},$$

(34) $$\langle 0|J_{A∥}^{\gamma d}|\phi^{d^{\prime }}(p)\rangle =if_{{}_{P}}^{d}\;p^{\gamma }\delta^{dd^{\prime }},$$

(35) $$\langle 0|J_{A⊥}^{\rho a}|A^{a^{\prime }}(p,\varepsilon )\rangle =f_{{}_{A}}\;\varepsilon^{\prime \;\rho }\;\delta^{aa^{\prime }},$$

(36) $$\langle 0|J_{V∥}^{\gamma d}|{\overset{˜}{\phi }}^{d^{\prime }}(p)\rangle =if_{{}_{S}}^{d}\;p^{\gamma }\delta^{dd^{\prime }},$$

where, *ε* and $\varepsilon^{\prime }$ denote the polarization vector of the vector meson and axial-vector meson respectively. By considering the mentioned items, the following relationships can be calculated:

(37) 〈B⁎(p1,ε1)|B⁎(p2,ε2)P(p3)〉=Λ(B⁎B⁎P)ε1μ⁎ε2νpα3p32Iˆ(〈0|T{JV⊥μa(x)JV⊥νb(0)JA∥αc(w)}|0〉),

(38) 〈B1(p1,ε′)|B⁎(p2,ε)P(p3)〉=Λ(B1B⁎P)εμ′⁎ενpα3p32Iˆ(〈0|{JA⊥μa(x)JV⊥νb(0)JA∥αc(w)}|0〉),

(39) $$\langle B_{1}(p_{1},\varepsilon_{1}^{\prime })|B_{1}(p_{2},\varepsilon_{2}^{\prime })\;V(p_{3},\varepsilon )\rangle =\Lambda (B_{1}\;B_{1}\;V)\varepsilon_{1\mu }^{\prime \;⁎}\;\varepsilon_{2\nu }^{\prime }\;\varepsilon_{\sigma }\;\overset{ˆ}{I}(\langle 0|T\left\{J_{A⊥}^{\mu a}(x)J_{A⊥}^{\nu b}(0)J_{V⊥}^{\sigma c}(w\right)\}|0\rangle ),$$

(40) 〈B1(p1,εA)|B⁎(p2,εV)S(p3)〉=Λ(B1B⁎S)εμ′⁎ενpα3p32Iˆ(〈0|{JA⊥μa(x)JV⊥νb(0)JV∥αc(w)}|0〉),

where, $\Lambda (O_{1}\;O_{2}\;O_{3})$ for the matrix element of $\langle O_{1}(p_{1})|O_{2}(p_{2})\;O_{3}(p_{3})\rangle$ is as:

(41) $$\Lambda (O_{1}\;O_{2}\;O_{3})=\frac{\left(p_{1}^{2}-m_{O_{1}}^{2}\right)}{f_{O_{1}}}\;\frac{\left(p_{2}^{2}-m_{O_{2}}^{2}\right)}{f_{O_{2}}}\;\frac{\left(p_{3}^{2}-m_{O_{3}}^{2}\right)}{f_{O_{3}}},$$

and the decay constant for the $i^{th}$ particle is denoted with $f_{O_{i}}$. Moreover,

(42) $$\overset{ˆ}{I}=\int d^{4}x\;d^{4}w\;e^{ip_{1}x-ip_{3}w},$$

is the double integration operator which is inserted after applying the Fourier transform. Using the following relations [46], [53] as:

(43) $$\phi^{a}(p,z)=\phi^{a}(p^{2},z)\frac{ip^{\alpha }}{p^{2}}A_{∥\alpha }^{0a}(p)\;,\;\;\;\;\;\;\;\;\;\;\;\;\;\;\;\;\;\pi^{a}(p,z)=\pi^{a}(p^{2},z)\frac{ip^{\alpha }}{p^{2}}A_{∥\alpha }^{0a}(p)\;,$$

(44) $$A_{⊥\mu }^{a}(q,z)=A^{a}(q^{2},z)\;A_{⊥\mu }^{0a}(q),\;\;\;\;\;\;\;\;\;\;\;\;\;\;\;\;\;\;V_{⊥\mu }^{b}(q,z)=V^{b}(q^{2},z)\;V_{⊥\mu }^{0b}(q)\;,$$

(45) $$V_{z}^{b}(q,z)=-\partial_{z}{\overset{˜}{\pi }}^{b}(q^{2},z)\;\frac{iq^{\alpha }}{q^{2}}V_{∥\alpha }^{0b}(q)\;,\;\;\;\;\;\;\;\;\;\;\;\;\;\;\;\;\;\partial^{\mu }\to -i{\left(\text{relevant}\;\text{momentum}\right)}^{\mu }\;,$$

and applying the limit $p_{i}^{2}\to m_{O_{i}}^{2}$ for (i=1,2,3) in the final results, the couplings of $\left(B^{⁎},B^{⁎},P\right)$, $\left(B_{1},B^{⁎},P\right)$, $\left(B_{1},B_{1},V\right)$, and $\left(B_{1},B^{⁎},S\right)$ vertices can be expressed as follows:

(46) $$g_{{}_{B^{⁎}\;B^{⁎}\;P}}=g_{5}^{3}\int_{0}^{z_{0}}dz\;\frac{k^{abc}}{M\;z^{3}}\left(\psi_{{}_{V}}^{a}(z)\;\psi_{{}_{V}}^{b}(z)\;\pi^{c}(z)\right),$$

(47) $$g_{{}_{B_{1}\;B^{⁎}\;P}}=g_{5}\;\int_{0}^{z_{0}}dz\;(\frac{f^{abc}}{2\;z}\;[\psi_{{}_{A}}^{a}(z)\;\psi_{{}_{V}}^{b}(z)\phi^{c}(z)\;]+\frac{g_{5}^{2}\;g^{abc}}{M\;z^{3}}\;\left[\psi_{{}_{A}}^{a}(z)\;\psi_{{}_{V}}^{b}(z)\pi^{c}(z)\right])+g_{5}^{3}\;\int_{0}^{z_{0}}dz\;(\frac{h^{abc}}{M\;z^{3}}\;\left[\psi_{{}_{A}}^{a}(z)\;\psi_{{}_{V}}^{b}(z)\pi^{c}(z)\right]),$$

(48) $$g_{{}_{B_{1}\;B_{1}\;V}}=g_{5}\;\int_{0}^{z_{0}}dz\;(\frac{f^{abc}}{z}\;\left[\psi_{{}_{A}}^{a}(z)\;\psi_{{}_{V}}^{b}(z)\psi_{{}_{A}}^{c}(z\right)]),$$

(49) $$g_{{}_{B_{1}\;B^{⁎}\;S}}=g_{5}\;\int_{0}^{z_{0}}dz\;(\frac{f^{abc}}{z}\;\left[\psi_{{}_{A}}^{a}(z)\;\psi_{{}_{V}}^{b}(z)\;\xi^{c}(z\right)]+\frac{f^{abc}}{M\;z}[\partial_{z}\psi_{{}_{A}}^{a}\;\psi_{{}_{V}}^{b}\;\partial_{z}{\overset{˜}{\pi }}^{c}-\partial_{z}\psi_{{}_{V}}^{a}\;\psi_{{}_{A}}^{b}\;\partial_{z}{\overset{˜}{\pi }}^{c}]).$$

In the previous relations, M is defined as:

(50) $$M=p_{1}^{2}+p_{3}^{2}-p_{2}^{2}.$$

**Figure 2:**
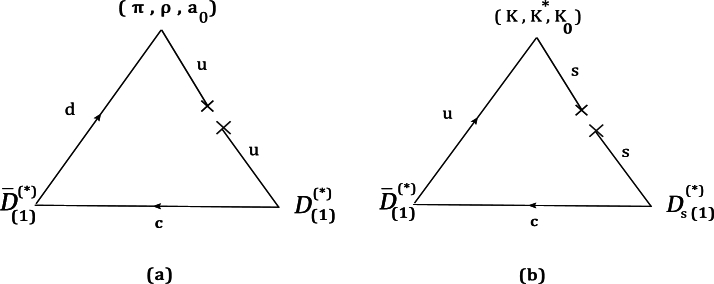
3-particle diagrams for (*D*^⁎^,*D*^⁎^,*P*), (*D*_1_,*D*^⁎^,*P*), (*D*_1_,*D*_1_,*V*) and (*D*_1_,*D*^⁎^,*S*) vertices. Diagram (a): $\left({\overset{¯}{D}}^{{}_{⁎0}},D^{{}_{⁎-}},\pi^{-}\right)$, $\left({\overset{¯}{D}}^{{}_{⁎0}},D_{1}^{{}_{-}},\pi^{-}\right)$, $\left({\overset{¯}{D}}_{1}^{{}_{0}},D_{1}^{{}_{-}},\rho^{-}\right)$, $\left({\overset{¯}{D}}^{{}_{⁎0}},D_{1}^{{}_{-}},a_{0}^{-}\right)$. Diagram (b): $\left({\overset{¯}{D}}^{{}_{⁎0}},D_{s}^{{}_{⁎-}},K^{+}\right)$, $\left({\overset{¯}{D}}^{{}_{⁎0}},D_{s1}^{{}_{-}},K^{+}\right)$, $\left({\overset{¯}{D}}_{1}^{{}_{0}},D_{s1}^{{}_{-}},K^{⁎+}\right)$, $\left({\overset{¯}{D}}^{{}_{⁎0}},D_{s1}^{{}_{-}},K_{0}^{+}\right)$.

To estimate couplings of $\left(D^{⁎},D^{⁎},P\right)$, $\left(D_{1},D^{⁎},P\right)$, $\left(D_{1},D_{1},V\right)$, and $\left(D_{1},D^{⁎},S\right)$ vertices, we make the replacements of B→D, $B^{⁎}\to D^{⁎}$ and $B_{1}\to D_{1}$ in Eqs. (46)-(49). These vertices are displayed in Fig. 2 (a,b). In this figure, part(a) shows the vertices of $\left({\overset{¯}{D}}^{{}_{⁎0}},D^{{}_{⁎-}},\pi^{-}\right)$, $\left({\overset{¯}{D}}^{{}_{⁎0}},D_{1}^{{}_{-}},\pi^{-}\right)$, $\left({\overset{¯}{D}}_{1}^{{}_{0}},D_{1}^{{}_{-}},\rho^{-}\right)$ and $\left({\overset{¯}{D}}^{{}_{⁎0}},D_{1}^{{}_{-}},a_{0}^{-}\right)$, and part(b) describes the vertices of $\left({\overset{¯}{D}}^{{}_{⁎0}},D_{s}^{{}_{⁎-}},K^{+}\right)$, $\left({\overset{¯}{D}}^{{}_{⁎0}},D_{s1}^{{}_{-}},K^{+}\right)$, $\left({\overset{¯}{D}}_{1}^{{}_{0}},D_{s1}^{{}_{-}},K^{⁎+}\right)$, and $\left({\overset{¯}{D}}^{{}_{⁎0}},D_{s1}^{{}_{-}},K_{0}^{+}\right)$.

## 4 Numerical analysis

This section reports numerical estimations for the masses, decay constants, wavefunctions of *B* mesons, and the couplings of vertices, as mentioned earlier. To achieve this goal, the values of $z_{0}$, $m_{q}$ and $\sigma_{q}$ for q=(u,d,s,c,b) are needed. For $N_{f}=4$, these values are fitted to the experimental masses of $\rho^{0}$, $\rho^{-}$, $a_{1}^{-}$, $\pi^{0}$, $\pi^{-}$, $K^{-}$, $K^{{}_{⁎-}}$, $D^{-}$ and $D^{{}_{⁎-}}$ mesons in reference [69]. The best global fit in MeV has been reported as $z_{0}^{-1}=(323\pm 1)$, $m_{u}=(8.5\pm 2.5)$, $m_{d}=(12.36\pm 2.45)$, $m_{s}=(195.31\pm 5.89)$ and $m_{c}=(1590.56\pm 8.42)$. Moreover, for the quark condensates in $\mathrm{Me}V^{3}$ the fitted values $\sigma_{u}={\left(173.65\pm 2.21\right)}^{3}$, $\sigma_{d}={\left(177.42\pm 3.15\right)}^{3}$, $\sigma_{s}={\left(226.20\pm 3.72\right)}^{3}$ and $\sigma_{c}={\left(310.35\pm 5.65\right)}^{3}$ are also calculated in reference [69]. To extend the model to $N_{f}=5$, it is necessary to know the values of $m_{b}$ and $\sigma_{b}$, which according to the experimental data reported in reference [78], for $m_{B^{{}_{{}_{-}}}}=(5279.34\pm 0.12)$ and $m_{B^{{}_{{}_{⁎0}}}}=(5324.71\pm 0.21)$ in MeV, the values of $m_{b}$ and $\sigma_{b}$ are estimated as $m_{b}=(4120\pm 5)\;\text{MeV}$ and $\sigma_{b}={\left(420\pm 1.12\right)}^{3}\;\mathrm{Me}V^{3}$. Note that the experimental value for *b* quark mass is $m_{b}=(4.18_{-0.02}^{+0.03})\;\text{GeV}$ [78]. Since the regions of uncertainty for experimental values of $m_{B^{{}_{{}_{-}}}}$ and $m_{B^{{}_{{}_{⁎-}}}}$ are small, the fitted values for $m_{b}$ and $\sigma_{b}$ also change in a minimal uncertainty interval. Now, having all of the required parameters of the model in hand, we can predict mass, decay constant, and wavefunction for bottom mesons and complete the Hard wall AdS/QCD model up to $N_{f}=5$. Predictions for the masses of $B^{{}_{0}},B_{s}^{{}_{0}},B_{c}^{{}_{-}},\eta_{b},B_{s}^{⁎0},B_{c}^{{}_{⁎-}},B_{1s}^{{}_{0}},B_{1c}^{{}_{-}},B_{1}^{{}_{-}}$, and $\chi_{{}_{b1}}$ are presented in Table 2. In this table, the measured masses for $B^{{}_{0}},B_{s}^{{}_{0}},B_{c}^{{}_{-}},\eta_{b},B_{s}^{⁎0},B_{1s}^{{}_{0}},B_{1}^{{}_{-}}$ and $\chi_{{}_{b1}}$ mesons are collected from [78]. For the cases mentioned in Table 1, if the value of ${M_{V}}^{2}$ for each state is equal to zero, such as wavefunction equations of ϒ and $\chi_{{}_{b0}}$ states, then the proposed model can not find mass values.

**Table 2:** Predicted and measured [78] masses for the pseudoscalar, vector, and axial vector *B* mesons. $B_{c}^{{}_{⁎-}}$ and $B_{1c}^{{}_{-}}$ states have not yet been observed in the laboratory.

Meson	Measured Mass (MeV)	This work (MeV)	Meson	Measured Mass (MeV)	This work (MeV)
$B^{{}_{0}}$	5279.66 ± 0.12	5280.17 ± 2.65	$B_{c}^{{}_{⁎-}}$	–	6510.14 ± 13.32
$B_{s}^{{}_{0}}$	5366.92 ± 0.10	5375.42 ± 7.28	$B_{1}^{{}_{-}}$	$5725.90_{-2.7}^{+2.5}$	5741.25 ± 3.42
$B_{c}^{{}_{-}}$	6274.47 ± 0.32	6312.65 ± 10.26	$B_{1s}^{{}_{0}}$	5828.70 ± 0.20	5892.11 ± 9.23
*η*_*b*_	9398.70 ± 2	9279.68 ± 4.71	$B_{1c}^{{}_{-}}$	–	6852.41 ± 10.54
$B_{s}^{⁎0}$	$5415.4_{-1.5}^{+1.8}$	5398.32 ± 8.16	$\chi_{{}_{b1}}$	9892.78 ± 0.57	9827.53 ± 4.67

**Figure 3:**
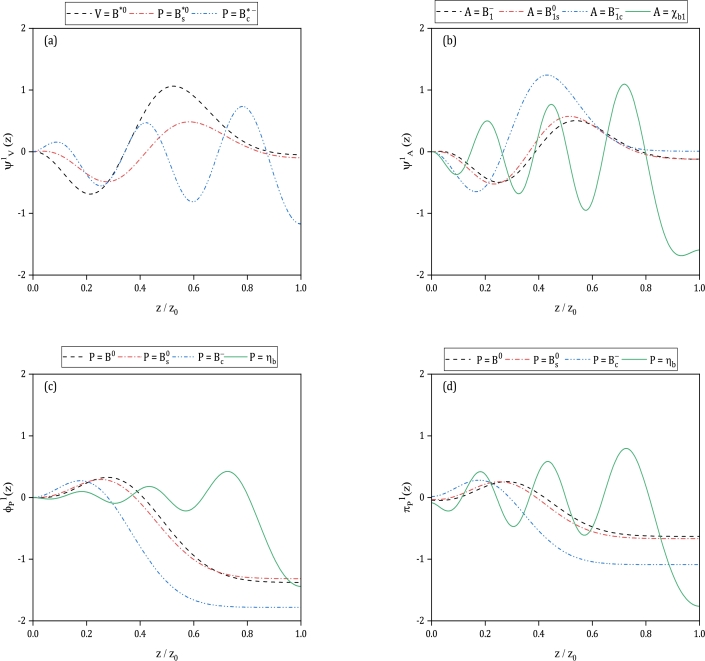
Wave functions of $\psi_{V}^{1}(z)$ (part(a)), $\psi_{A}^{1}(z)$ (part(b)), $\phi_{P}^{1}(z)$ (part(c)), and $\pi_{P}^{1}(z)$ (part(d)) for $V=(B^{{}_{0⁎}},B_{s}^{⁎0},B_{c}^{{}_{⁎-}})$, $A=(B_{1}^{{}_{-}},B_{1s}^{{}_{0}},B_{1c}^{{}_{-}},\chi_{{}_{b1}})$ and $P=(B^{{}_{0}},B_{s}^{{}_{0}},B_{c}^{{}_{-}},\eta_{b})$ in terms of *z*/*z*_0_. Superscript (1) in these wave functions, indicates that they are plotted for the ground state mesons. Differential equations and boundary conditions for *ψ*_*V*_(*z*), *ψ*_*A*_(*z*), *ϕ*_*P*_(*z*) and *π*_*P*_(*z*) are explained in Sec. 2 and masses are given in Table 2.

We can now calculate the wave functions for the studied mesons according to the calculated masses. The wave functions $\psi_{V}^{1}$, $\psi_{A}^{1}$, $\phi_{P}^{1}$, and $\pi_{P}^{1}$ with $V=(B^{{}_{0⁎}},B_{s}^{⁎0},B_{c}^{{}_{⁎-}})$, $A=(B_{1}^{{}_{-}},B_{1s}^{{}_{0}},B_{1c}^{{}_{-}},\chi_{{}_{b1}})$ and $P=(B^{{}_{0}},B_{s}^{{}_{0}},B_{c}^{{}_{-}},\eta_{b})$ are plotted for the ground state mesons as functions of $z/z_{0}$ ratio in Fig. 3 (a,b,c,d). In this figure, the wavefunctions of $B^{{}_{⁎0}}$, $B_{1}^{-}$ and $B^{0}$ are plotted with dash lines, while for the wavefunctions of bottom-strange mesons $B_{s}^{⁎0}$, $B_{1s}^{{}_{0}}$ and $B_{s}^{{}_{0}}$ dash-dotted lines are utilized. The bottom-charm mesons $B_{c}^{{}_{⁎-}}$, $B_{1c}^{{}_{-}}$, and $B_{c}^{{}_{-}}$ are displayed with dash-dot-dot lines and the plots of $\chi_{{}_{b1}}$ and $\eta_{b}$ are presented with solid lines.

In this step, the results of numerical analysis for the decay constant of the vector, axial vector, and pseudoscalar *B* mesons are presented in Table 3. Predictions for the decay constant of $B^{0}$, $B_{s}^{{}_{0}}$, $B_{c}^{{}_{-}}$, $B^{{}_{0⁎}}$, $B_{s}^{⁎0}$, $B_{c}^{{}_{⁎-}}$, $B_{1}^{{}_{-}}$, $B_{1s}^{{}_{0}}$ and $B_{1c}^{{}_{-}}$ mesons are listed in this Table. Other reported predictions, such as LQCD [79], [80], QCDSR [81], [82], [83], [84], [85], [86], [87], [88], Light Front Quark model (LFQM) [89], [90], [91], [92], [93], AdS/LF QCD [94], relativistic Bethe-Salpeter (BS) model (BS) [95], [96], [97], Quark model (QM) [98], [99], [100] and Constituent Quark model (CQM) [101], for decay constants of considered mesons are also included in this Table. For this group of mesons, experimental measurements have been done only for decay constant $B^{0}$ as $f_{B^{0}}=(188\pm 25)\;\text{MeV}$ [78]. Using the values listed in Table 3, the estimation for the ratio of vector to pseudoscalar decay constant yields:

(51) $$\frac{F_{B^{{}_{0⁎}}}}{f_{B^{0}}}=1.06\pm 0.04\;,$$

(52) $$\frac{F_{B_{s}^{{}_{⁎0}}}}{f_{B_{s}^{{}_{0}}}}=1.09\pm 0.06\;,$$

(53) $$\frac{F_{B_{c}^{{}_{⁎-}}}}{f_{B_{c}^{{}_{-}}}}=1.02\pm 0.05\;.$$

**Figure 4:**
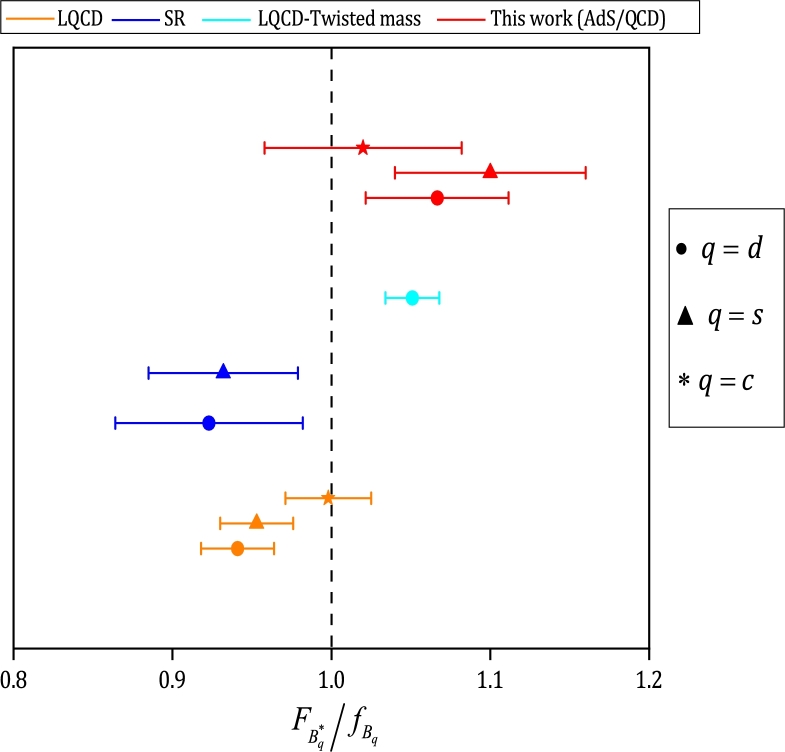
Comparison for the ratio of vector to pseudoscalar decay constants for $B^{{}_{0⁎}}/B^{0}$ (filled circles), $B_{s}^{{}_{⁎0}}/B_{s}^{{}_{0}}$ (filled triangles) and $B_{c}^{{}_{⁎-}}/B_{c}^{{}_{-}}$ (filled asterisks). The top three lines (in red) are the estimated values. The results of LQCD [79], (SR) [102] and LQCD with twisted mass quarks [103] are shown in orange, blue and cyan, respectively. The black dashed line marks the value 1.0.

It is noteworthy that according to the notation used in Sec. 2, $F_{V}=f_{V}^{1/2}$. The values obtained from the calculations are compared with the results of LQCD [79], Sum Rules (SR) [102] and LQCD with twisted mass quarks [103] in Fig. 4, and the values of $\frac{F_{B_{q}^{{}_{⁎}}}}{f_{B_{q}}}$ circle, triangle, and asterisk symbols are shown for q=d,s and *c*, respectively. Moreover, the value 1.0 is also marked with a black dashed line.

**Table 3:** Comparison of results from different models for the decay constants of *B*^0^, $B_{s}^{{}_{0}}$, $B_{c}^{{}_{-}}$, $B^{{}_{0⁎}}$, $B_{s}^{⁎0}$, $B_{c}^{{}_{⁎-}}$, $B_{1}^{{}_{-}}$, $B_{1s}^{{}_{0}}$, and $B_{1c}^{{}_{-}}$ mesons in MeV.

State	LQCD [79], [80]	QCDSR [81], [82], [83], [84], [85], [86], [87], [88]	LFQM [89], [90], [91], [92], [93]	AdS/LF QCD [94]	BS [95], [96], [97]	QM [98], [99], [100]	CQM [101]	This work
*B*^0^	187.1 ± 0.42	204.00 ± 0.51	204 ± 31	$193.40_{-10.60}^{+4.70}$	196 ± 29	155 ± 15	189	190 ± 8
$B_{s}^{{}_{0}}$	227.20 ± 3.40	234.50 ± 4.40	281 ± 54	$225.5_{-7.20}^{+6.20}$	216 ± 32	210 ± 20	218	205 ± 10
$B_{c}^{{}_{-}}$	434 ± 15	528 ± 19	406	−	322 ± 42	400 ± 40	−	420 ± 17
$B^{{}_{0⁎}}$	185.90 ± 7.20	210 ± 6	225.20 ± 38	$198.7_{-11.30}^{+4.90}$	238 ± 18	221	219	202 ± 7
$B_{s}^{{}_{⁎0}}$	223.10 ± 5.40	221 ± 7	313 ± 67	$231.90_{-7.60}^{+6.60}$	272 ± 20	259	251	228 ± 15
$B_{c}^{{}_{⁎-}}$	428 ± 14	415 ± 31	436	−	418 ± 24	−	−	427 ± 18
$B_{1}^{{}_{-}}$	173 ± 23	555 ± 201	175	−	$266.60_{-9.00}^{+8.80}$	−	−	195 ± 9
$B_{1s}^{{}_{0}}$	200 ± 20	572 ± 213	183	−	$286.10_{-7.50}^{+7.10}$	−	−	218 ± 12
$B_{1c}^{{}_{-}}$	−	−	157	−	$227.00_{-12.70}^{+13.40}$	−	−	235 ± 17

**Figure 5:**
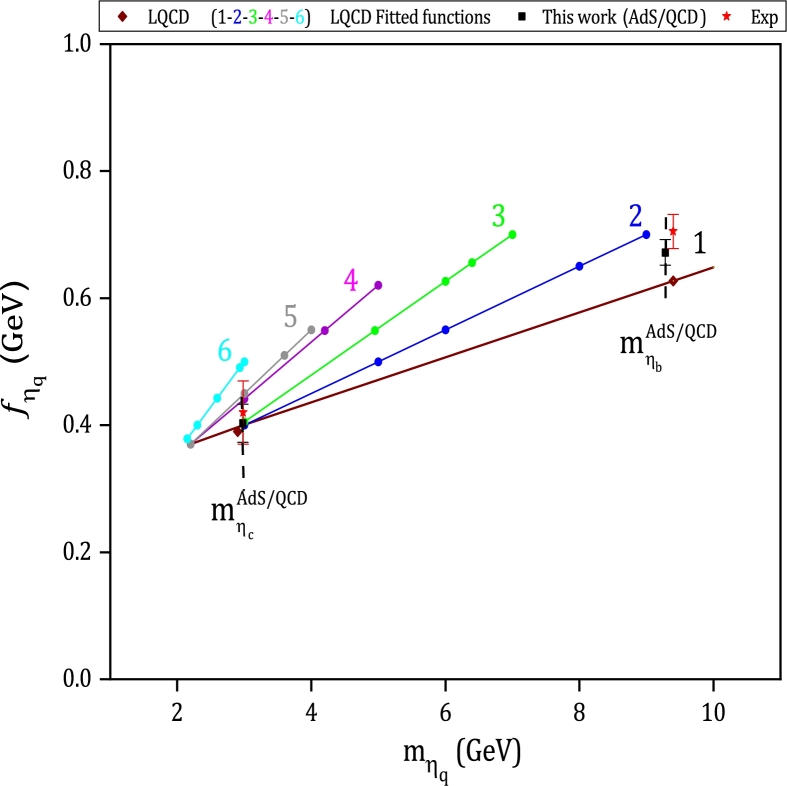
Results for the pseudoscalar heavyonium decay constants as a function of their masses for experimental measurements (in asterisk symbol) [105], LQCD (in diamond symbol), and LQCD fitted functions (in circle symbol) [104]. The studied results using Hard-Wall AdS/QCD model are shown with square symbol.

According to the proposed model, we can also study the pseudoscalar heavyonium mesons. Considering the mass value of $m_{\eta_{c}}=(2979.62\pm 2.43)\;\text{MeV}$ for the $\eta_{c}$ meson [69], the decay constant of this state is calculated as $f_{\eta_{c}}=(402.86\pm 30)\;\text{MeV}$ and the value of $f_{\eta_{b}}=(672.95\pm 20)\;\text{MeV}$ is also estimated for $\eta_{b}$. Compared to other works, LQCD predicts $f_{\eta_{c}}=(394\pm 22)\;\text{MeV}$ and $f_{\eta_{b}}=(667\pm 8)\;\text{MeV}$ [104], and using experimental decay widths of $J/\psi \to e^{+}\;e^{-}$ and $ϒ\to e^{+}\;e^{-}$ mentioned in [105], the value of $\eta_{c}$ and $\eta_{b}$ are reported as $f_{\eta_{c}}=(420\pm 52)\;\text{MeV}$ and $f_{\eta_{c}}=(705\pm 27)\;\text{MeV}$. All the results of $f_{\eta q}$ including the main results and the fitted functions of LQCD [104], experimental values [105], and the presented model are plotted versus $m_{\eta_{q}}$ for q=(c,b) in Fig. 5. As can be found from this figure, our predictions are placed in the primary LQCD fitted function range.

**Figure 6:**
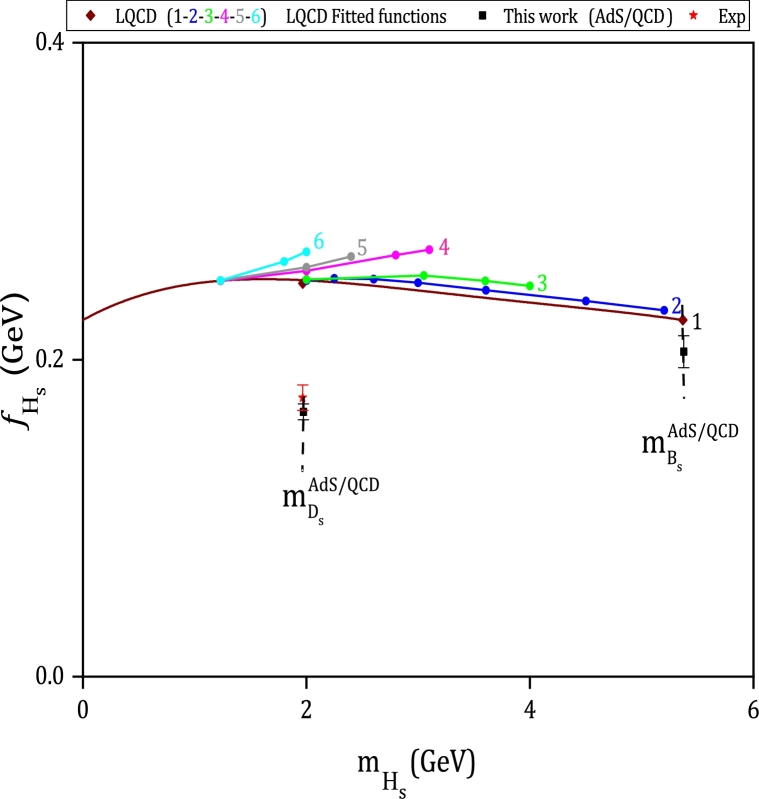
Leptonic decay constants $f_{H_{s}}$ for pseudoscalar states *H*_*s*_ as a function of $m_{H_{s}}$ for presented results, LQCD fitted functions [104], and AdS/QCD (for *H* = *D*) [69]. Experimental data for $f_{D_{s}}$, $m_{D_{s}}$ and $m_{B_{s}}$ can be found in [78].

The leptonic decay constants $f_{H_{s}}$ for the pseudoscalar heavy-strange mesons with quark contents $h\overset{¯}{s}$ are also plotted as a function of their masses in Fig. 6. The decay constant and mass of $D_{s}$ meson for AdS/QCD model are taken from reference [69] as $f_{D_{s}}=(167.66\pm 4.85)\;\text{MeV}$ and $m_{D_{s}}=(1972.63\pm 2.37)\;\text{MeV}$. Experimental values of these observables [78], the main results and the fitted functions of LQCD [104] are also included in this figure. From this figure, we found that the calculated result of Hard-Wall AdS/QCD for $D_{s}$ is in good agreement with the experimental data, and considering the uncertainty region, our result is within the range of LQCD estimation for $B_{s}$.

The ratio of vector to pseudoscalar decay constants multiplied by the ratio of the square root of the masses for meson $B_{q}$ with q=(u,d,s) is defined as:

(54) $$R_{q}=\frac{F_{H^{⁎}}\sqrt{m_{H^{⁎}}}}{f_{H}\sqrt{m_{H}}}$$

**Figure 7:**
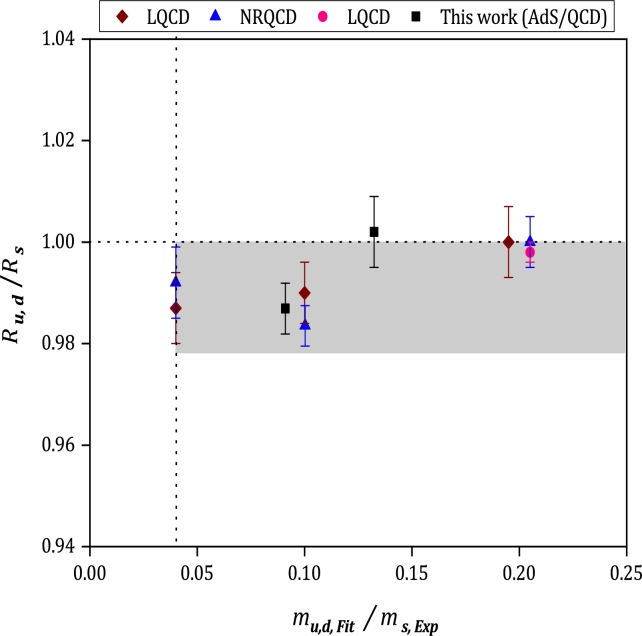
Results for the ratio of *R*_*u*,*d*_/*R*_*s*_ versus *m*_*u*,*d*,Fit_/*m*_*s*,Exp_. The calculated results are shown with square symbols, and the other estimations reported in [79] are displayed with diamond, triangle, and circle symbols. The gray-shaded region is a physical band including all systematic uncertainties calculated from LQCD in [79].

**Figure 8:**
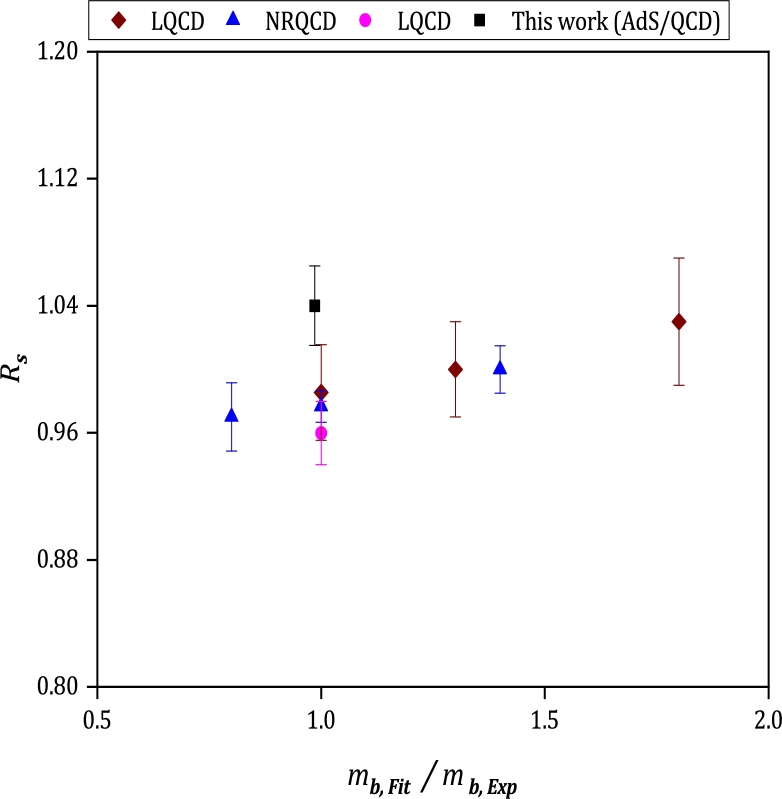
Comparison for the ratio of *R*_*s*_ in studied model, LQCD and NRQCD of [79].

The calculated results of $R_{u,d}/R_{s}$, are plotted in terms of the light quark mass (u,d) in units of the physical *s* quark mass in Fig. 7. The predictions of LQCD and NonRelativistic QCD (NRQCD) with their uncertainty regions from reference [79] are also shown in this figure. The gray shaded band in this figure gives the physical results of LQCD, including all systematic errors, which is obtained in [79]. Considering the lower bound of our estimation for $R_{u,d}/R_{s}$, the obtained value is placed in the range of physical results derived from LQCD. For better understanding, the values of $R_{s}$ are also plotted versus $m_{b,\mathrm{Fit}}/m_{b,\mathrm{Exp}}$ in Fig. 8. For comparison, the reported results of LQCD and NRQCD [79] are added in this figure.

To estimate the values of $g_{{}_{B^{⁎}\;B^{⁎}\;P}}$, $g_{{}_{B_{1}\;B^{⁎}\;P}}$, $g_{{}_{B_{1}\;B_{1}\;V}}$ and $g_{{}_{B_{1}\;B^{⁎}\;S}}$, Table 4, which includes masses, is used in these calculations. From the set of masses listed in this Table, the values of $m_{\rho^{-}}$, $m_{K^{{}_{⁎-}}}$, $m_{\pi^{-}}$, $m_{K^{-}}$ and $m_{D^{{}_{⁎-}}}$ are related to experimental values, which are reported in [78]. The other masses are extracted from Hard-Wall AdS/QCD model described in [69], [70]. Moreover, the nonzero values for $g^{abc}$, $h^{abc}$, and $k^{abc}$, which are used in numerical analysis are calculated and presented in Table 5. The final results of estimations for couplings of ($B^{⁎},B^{⁎},P$), ($B_{1},B^{⁎},P$), ($B_{1},B_{1},V$), and ($B_{1},B^{⁎},S$) vertices and couplings of ($D^{⁎},D^{⁎},P$), ($D_{1},D^{⁎},P$), ($D_{1},D_{1},V$), and ($D_{1},D^{⁎},S$) vertices are reported in Table 6 and Table 7, respectively. Table 6, Table 7 also compare the presented strong couplings, results of the 3PSR model [1], [12], [15], [21], [23], and the LCSR approach. In the 3PSR model, the correlation function is usually calculated regarding the QCD degrees of freedom, such as quark and gluon condensate. In the LCSR approach [27], [106], the strong couplings are derived in terms of the light-cone distribution amplitudes (LCDAs), including twist functions. These two models look for suitable regions of threshold and Borel parameters to suppress contributions from higher states. In this study, couplings are obtained based on particle wave functions and coefficients $h^{abc}$, $g^{abc}$, and $k^{abc}$. The main parameters for wave functions and coefficients are calculated by fitting the experimental values of masses and decay constants to the presented model predictions.

**Table 4:** Meson masses of strong couplings. The experimental masses of *ρ*^−^, $K^{{}_{⁎-}}$, *π*^−^, *K*^−^ and $D^{{}_{⁎-}}$ are reported in [78] and masses of $a_{0}^{-}$, $K_{0}^{{}_{+}}$, $D^{{}_{⁎0}}$, $D_{s}^{{}_{⁎-}}$, $D_{1}^{{}_{0}}$, $D_{s1}^{{}_{-}}$, and $D_{1}^{{}_{-}}$ are estimated using Hard-Wall AdS/QCD in [69], [70].

Meson	Mass (MeV)	Meson	Mass (MeV)	Meson	Mass (MeV)
*ρ*^−^	775.49 ± 0.34	$K^{{}_{⁎-}}$	891.66 ± 0.26	$a_{0}^{-}$	985.50 ± 4.52
$K_{0}^{{}_{+}}$	1402 ± 5.20	$D^{{}_{⁎0}}$	2005.53 ± 6.65	$D_{s}^{{}_{⁎-}}$	2122.90 ± 9.42
$D_{1}^{{}_{0}}$	2423.62 ± 4.52	$D_{s1}^{{}_{-}}$	2461.50 ± 5.42	$D_{1}^{{}_{-}}$	2427.25 ± 3.28
$\pi^{{}_{-}}$	139.75 ± 0.00	$K^{{}_{+}}$	493.67 ± 0.01	$D^{{}_{⁎-}}$	2010.26 ± 0.05

**Table 5:** Used values for *g*^*abc*^, *h*^*abc*^, *l*^*abc*^ and *k*^*abc*^ in numerical analysis with $v_{q}(z)=\zeta m_{q}z+\frac{1}{\zeta }\sigma_{q}z^{3}$ and *q* = (*u*,*d*,*s*,*c*,*b*).

(*a*,*b*,*c*)	*g*^*abc*^	*h*^*abc*^	*k*^*abc*^
(1,11,15)	$\frac{1}{2}(v_{d}+v_{u})(v_{u}+v_{b})$	$\frac{1}{2}(v_{u}-v_{d})(v_{d}+v_{b})$	$\frac{1}{2}(v_{d}+v_{u})(v_{d}-v_{b})$
(4,12,20)	$\frac{1}{2}(v_{u}+v_{s})(v_{s}+v_{b})$	$\frac{1}{2}(v_{u}-v_{s})(v_{s}+v_{b})$	$\frac{1}{2}(v_{s}-v_{b})(v_{u}+v_{s})$
(1,9,13)	$\frac{1}{2}(v_{u}+v_{d})(v_{u}+v_{c})$	$\frac{1}{2}(v_{u}-v_{d})(v_{d}+v_{c})$	$\frac{1}{2}(v_{u}+v_{d})(v_{d}-v_{c})$
(4,9,17)	$\frac{1}{2}(v_{u}+v_{s})(v_{u}+v_{c})$	$\frac{1}{2}(v_{u}-v_{s})(v_{s}+v_{c})$	$\frac{1}{2}(v_{s}-v_{c})(v_{u}+v_{s})$

**Table 6:** Strong coupling constants of (*B*^⁎^,*B*^⁎^,*P*), (*B*_1_,*B*^⁎^,*P*), (*B*_1_,*B*_1_,*V*), and (*B*_1_,*B*^⁎^,*S*) vertices with the corresponding errors in studied model (AdS/QCD), 3PSR [15], and LCSR approach [27], [106].

Vertex	3PSR [15]	LCSR [27], [106]	AdS/QCD
$\left({\overset{¯}{B}}^{{}_{⁎0}},B^{{}_{⁎+}},\pi^{-}\right)$	−	−	15.27 ± 2.56
$\left(B^{{}_{⁎+}},B_{s}^{{}_{⁎0}},K^{+}\right)$	−	−	13.46 ± 2.18
$\left({\overset{¯}{B}}_{1}^{{}_{0}},B^{{}_{⁎+}},\pi^{-}\right)$	54.01 ± 15.51	56 ± 15	19.92 ± 3.12
$\left(B_{1}^{{}_{⁎+}},B_{s}^{{}_{⁎0}},K^{+}\right)$	−	−	17.65 ± 3.02
$\left({\overset{¯}{B}}_{1}^{{}_{0}},B_{1}^{{}_{+}},\rho^{-}\right)$	−	0.57 ± 0.04	1.89 ± 0.31
$\left({\overset{¯}{B}}_{1}^{{}_{+}},B_{s1}^{{}_{0}},K^{⁎+}\right)$	−	0.66 ± 0.04	1.62 ± 0.29
$\left({\overset{¯}{B}}_{1}^{{}_{0}},B^{{}_{⁎+}},a_{0}^{-}\right)$	−	−	5.24 ± 0.98
$\left({\overset{¯}{B}}_{1}^{{}_{+}},B_{s}^{{}_{⁎0}},K_{0}^{+}\right)$	−	−	4.39 ± 0.71

**Table 7:** Predictions for the strong couplings of (*D*^⁎^,*D*^⁎^,*P*), (*D*_1_,*D*^⁎^,*P*), (*D*_1_,*D*_1_,*V*) and (*D*_1_,*D*^⁎^,*S*) vertices and the results of 3PSR [1], [12], [15], [21], [23] and LCSR [27], [106], [107] methods.

Vertex	3PSR [1], [12], [15], [21], [23]	LCSR [27], [106], [107]	AdS/QCD
$\left({\overset{¯}{D}}^{{}_{⁎0}},D^{{}_{⁎-}},\pi^{-}\right)$	−	−	6.02 ± 1.85
$\left({\overset{¯}{D}}^{{}_{⁎0}},D_{s}^{{}_{⁎-}},K^{+}\right)$	5.61 ± 1.64	−	4.85 ± 1.62
$\left({\overset{¯}{D}}^{{}_{⁎0}},D_{1}^{{}_{-}},\pi^{-}\right)$	19.78 ± 3.32	23 ± 5	7.45 ± 1.96
$\left({\overset{¯}{D}}^{{}_{⁎0}},D_{s1}^{{}_{-}},K^{+}\right)$	1.72 ± 0.21	1.67 ± 0.57	2.35 ± 1.21
$\left({\overset{¯}{D}}_{1}^{{}_{0}},D_{1}^{{}_{-}},\rho^{-}\right)$	6.60 ± 0.31	2.96 ± 0.21	3.79 ± 1.25
$\left({\overset{¯}{D}}_{1}^{{}_{0}},D_{s1}^{{}_{-}},K^{⁎+}\right)$	4.22 ± 0.55	3.24 ± 0.23	3.21 ± 1.14
$\left({\overset{¯}{D}}^{{}_{⁎0}},D_{1}^{{}_{-}},a_{0}^{-}\right)$	−	−	4.12 ± 1.36
$\left({\overset{¯}{D}}^{{}_{⁎0}},D_{s1}^{{}_{-}},K_{0}^{+}\right)$	4.24 ± 0.42		3.85 ± 1.25

As seen from Table 6, Table 7, couplings of *B* mesons (including every combination from vector and axial vector states) to light mesons (including pseudoscalar, vector, and scalar) are more significant than corresponding couplings of *D* mesons. Our numeric calculations show that the most important factors are as follows:

• The value of $\sigma_{b}$ is greater than $\sigma_{c}$; therefore, the factors $g^{abc}$, $h^{abc}$, and $k^{abc}$ for vertices involving *B* mesons are greater than similar factors for vertices including *D* mesons. For example, in the range of $\varepsilon \leq z\leq z_{0}$, the ratio of $k_{\left({\overset{¯}{B}}^{{}_{⁎0}},B^{{}_{⁎+}},\pi^{-}\right)}^{abc}/k_{\left({\overset{¯}{D}}^{{}_{⁎0}},D^{{}_{⁎-}},\pi^{-}\right)}^{abc}$ changes from 1.40 to 1.55.
• As can be seen in Table 2, Table 4, mass values for *B* meson are also greater than for *D* meson. In addition, for 87% of the range of $\varepsilon \leq z\leq z_{0}$, the wavefunction value for *B* meson is greater than *D* meson wavefunction.

Considering these two factors, according to Table 6 and Table 7, it can be understood that the coupling values of *B* meson are greater than those of *D* meson.

## 5 Conclusion

In this study, we consider *B* meson spectroscopy and wavefunctions of these states using the hard-wall model of AdS/QCD, including five quark flavors as q=(u,d,s,c,b). To obtain $m_{b}$ and $\sigma_{b}$, we used the experimental masses of $B^{{}_{{}_{-}}}$ and $B^{{}_{{}_{⁎0}}}$ states, and calculated decay constant of pseudoscalar heavyonium mesons and the ratio of vector to pseudoscalar decay constants over the presented model. Our model's results and predictions were compared with those of other studies. The results obtained for the decay constant of pseudoscalar heavyonium mesons are in good agreement with the results presented by the LQCD model.

Finally, we extended the results of our model to the strong vertices of charmed and bottom mesons ($D_{\left(s\right)}^{⁎}$, $D_{(s)1}$, $B_{\left(s\right)}^{⁎}$, and $B_{(s)1}$) with light mesons (*π*, *ρ*, $a_{0}$, *K*, $K^{⁎}$, and $K_{0}$) and compared the obtained results with the reported results of 3PSR and LCSR methods.

## CRediT authorship contribution statement

**S. Momeni:** Conceptualization. **M. Saghebfar:** Writing – review & editing.

## Declaration of Competing Interest

The authors declare that they have no known competing financial interests or personal relationships that could have appeared to influence the work reported in this paper.

## Data Availability

All experimental data used in this study are described in the references [76], [105]. Also, other considered theoretical data are referenced in the text of each section, and the data from the output of this article are also mentioned in the Table 2, Table 3, Table 6, Table 7.
